# Examining the Role of Discrimination in Prenatal Care Utilization: A Systematic Review Using the Social‐Ecological Model

**DOI:** 10.1002/nur.70033

**Published:** 2025-11-16

**Authors:** Abdul‐Manaf Mutaru, Alexa Parra, Cynthia Nicole Lebron, Wonsuk Yoo, Hudson P. Santos

**Affiliations:** ^1^ School of Nursing and Health Studies University of Miami Coral Gables Florida USA

**Keywords:** health care disparities, maternal health services, prenatal care utilization, Social‐Ecological model

## Abstract

Timely and adequate prenatal care (PNC) is essential for optimizing maternal and infant health outcomes. However, persistent disparities in PNC utilization exist in the United States, particularly among ethnic/racial minority populations. While discrimination has been recognized as a barrier, its multifaceted influence across social and structural contexts remains underexplored. This systematic review, guided by the Social‐Ecological Model, synthesizes evidence on how various forms of discrimination affect PNC utilization. We conducted comprehensive searches in PubMed, Web of Science, and CINAHL for peer‐reviewed studies published in the United States between 2010 and 2024. After screening 342 records, 11 studies met the inclusion criteria. Five studies employed qualitative methods, five used quantitative methods, and one utilized a mixed‐methods approach. Findings revealed that structural discrimination, such as language barriers and institutional policies, was associated with delayed or insufficient PNC utilization. Interpersonal dynamics, including negative patient‐provider interactions and implicit bias, discouraged engagement with care. Additionally, intrapersonal factors, like internalized discrimination, shaped perceptions of care quality and trust in healthcare systems. The review highlights how discrimination operates across multiple levels to influence PNC behaviors and outcomes. Addressing discrimination requires culturally responsive care models, provider training in cultural humility, and institutional reforms aimed at equity. Future research should explore protective factors, such as social support and resilience, that may buffer the negative effects of discrimination. Understanding these dynamics is crucial for developing interventions that promote equitable and effective PNC utilization.

## 1 Introduction

Maternal and infant health outcomes remain urgent public health issues in the United States (U.S.), with high rates of maternal mortality (32.9 deaths per 100,000 live births), preterm birth (10.28%), and low birth weight (8.60%) (Osterman et al. 2024). These adverse outcomes disproportionately affect ethnically and racially minoritized populations, particularly non‐Hispanic Black women, who are three to four times more likely to die from pregnancy‐related causes than their White counterparts (Declercq and Zephyrin 2020; Girardi et al. 2023; Hoyert 2024; Perry et al. 2024). Ethnic and racial minority populations are defined in the U.S. to include American Indians (including Alaska Natives, Eskimos, and Aleuts); Asian Americans; Native Hawaiians and other Pacific Islanders; Black (African Americans); and Hispanics (United States 2010). The consequences of adverse birth outcomes extend beyond individual health, contributing to long‐term child health challenges, financial strain, and emotional hardship, while reinforcing systemic inequities in already marginalized communities.

Early, consistent, and quality prenatal care (PNC) utilization offers protection during pregnancy against adverse maternal‐infant outcomes (Gajate‐Garrido 2013; Salinas‐Miranda et al. 2020). PNC involves continuously monitoring pregnancy and designing care plans that address the medical, nutritional, psychosocial, cultural, and educational needs of pregnant women. These plans are regularly revised based on the stage of pregnancy, in collaboration with the healthcare provider (American Academy of Pediatrics & American College of Obstetricians and Gynecologists 2017). Prominent health organizations, including the American College of Obstetricians and Gynecologists (ACOG) and the World Health Organization (WHO), have recommended guidelines for PNC utilization (American Academy of Pediatrics & American College of Obstetricians and Gynecologists 2017; World Health Organization 2018). These organizations each recommend, on average, about 14 PNC visits for low‐risk pregnancies before full‐term delivery (39–40 weeks of pregnancy).

The objective of PNC is to enhance pregnant women's overall health through screening, identify threats to healthy pregnancies, and provide timely interventions. As such, the contribution of PNC to positive maternal and infant health outcomes is remarkable (Carter et al. 2016; Gardner et al. 2023). To illustrate, having four or fewer PNC visits before delivery is associated with maternal mortality (Gadson et al. 2017). Additionally, pregnancy‐related deaths among ethnic and racial minority women are linked to delayed initiation of PNC, including beginning care in the second trimester (14–26 weeks’ gestation) (Carter et al. 2016; Chen et al. 2025).

Notably, the U.S. has seen a decline in the initiation of PNC during the first trimester (0–13 weeks’ gestation), with rates dropping from 78.3% in 2021 to 77.0% in 2022 (Osterman et al. 2024). Even though the specific cause for this decline is unclear, several factors could be responsible, including social determinants of health (SDoH), referred to as the nonmedical factors and conditions in which people are born, grow, live, work, and age (Braveman and Gottlieb 2014; Guilamo‐Ramos et al. 2023). For instance, disparities in the timing of PNC have persisted across ethnic and racial minority groups compared to non‐Hispanic White women. Specifically, the percentage of women initiating first‐trimester PNC declined among Hispanic women (from 72.5% to 70.5%), Black women (from 69.7% to 67.6%), and Native Hawaiian and Other Pacific Islander women (from 64.8% to 49.7%). In contrast, the rate among non‐Hispanic White women remained relatively stable, decreasing only slightly from 83.2% to 82.6% (Osterman et al. 2024).

### 1.1 Barriers to Prenatal Care Utilization

Extant literature also documents the role of SDoH factors in the observed lower PNC utilization in the U.S. Late initiation or fewer PNC visits are particularly associated with low income (Meyer et al. 2016), lack of insurance (Lee et al. 2024), transportation costs (Deng et al. 2024), neighborhood safety, and unstable housing Wolf et al. 2024). Also, discrimination is recognized as an SDoH (Centers for Disease Control and Prevention 2024; Office of Disease Prevention and Health Promotion n.d). Studies (Capp 2022; Masters et al. 2024) have explored how discrimination is associated with adverse maternal and infant health outcomes, especially among ethnic and racial minority populations. However, to effectively mitigate poor maternal and child health outcomes, PNC also requires attention.

According to *Healthy People 2030*, discrimination is defined as a socially structured action that is unfair or unjustified and harms individuals and groups (U.S. Department of Health and Human Services n.d). Considering that discrimination occurs across many different axes of oppression, this review focuses on racial discrimination among ethnic and racial minorities in the U.S. Racial discrimination refers to treating someone unfairly or unequally because of their race. This can include negative treatment based on racial characteristics such as skin color, facial features, or hair texture, and may occur even when the individuals involved share the same racial identity (U.S. Equal Employment Opportunity Commission n.d). Research suggests that ethnic and racial minority populations, particularly Black pregnant women, frequently encounter high levels of discrimination during prenatal care (Prater et al. 2023). These repeated experiences of bias can erode trust in the healthcare system and contribute to preferences for racially concordant providers (those who share the same racial identity) as a means of seeking more respectful and culturally safe care (Teal et al. 2024). Racial concordance in provider‐patient relationships has also been associated with improved health outcomes, potentially due to increased communication, trust, and shared cultural understanding. As racial discrimination persists, it continues to create barriers to PNC utilization, contributing to persistent disparities in maternal and infant health outcomes.

### 1.2 Aim of the Review

The available evidence on the relationship between discrimination and PNC utilization in the U.S. is limited and sporadic. This underscores the need for a review to present a unified understanding guided by a framework. A harmonized understanding of how discrimination influences PNC utilization is crucial, as it may help explain the disproportionate rates of poor maternal and infant health outcomes. To our knowledge, no current review specifically addresses the relationship between discrimination and PNC utilization among ethnic and racial minority populations in the U.S. This systematic review employed the Social‐Ecological Model (SEM) as a conceptual framework to synthesize findings from existing studies and examine the relationship between discrimination experiences and PNC utilization among ethnic and racial minority populations in the U.S. This structured approach provides a nuanced understanding of the effect of discrimination on PNC utilization among these populations as a possible contributor to health disparities.

## 2 Methods

### 2.1 Discrimination from the Social‐Ecological Perspective

This review's theoretical framework is rooted in the Social‐Ecological Model (Bronfenbrenner 1979), a multidimensional paradigm that elucidates the complex interplay of factors influencing health behaviors and outcomes. Using this model, we emphasize the distinct dynamics operating across multiple levels of influence, including intrapersonal, interpersonal, community, and structural, that collectively shape PNC utilization.

This framework, therefore, presents discrimination in different forms at various levels: intrapersonal, interpersonal, community, and societal/structural. At the intrapersonal level, we considered studies that examined how individual‐level factors, including perceived discrimination, avoiding care due to racism, and internalized discrimination, affect PNC utilization, mainly through qualitative interviews. Similarly, studies grouped under the interpersonal level reported how negative patient‐provider interactions and providers’ implicit and explicit bias affect PNC utilization, also through qualitative methods. The community level included studies that presented how everyday discrimination experiences affected PNC. Studies capturing this usually measured it using the *Daily Life Experiences of Racism and Bother Scale (DLERBS), Perceived Discrimination Scale, or Racism and Life Experience Index (RLEI)* (Harrell 2012; Williams et al. 1997). Similarly, the structural level encompasses system‐level factors, including policies or structures that inherently exclude or are reported to hinder PNC utilization.

**Figure 1:**
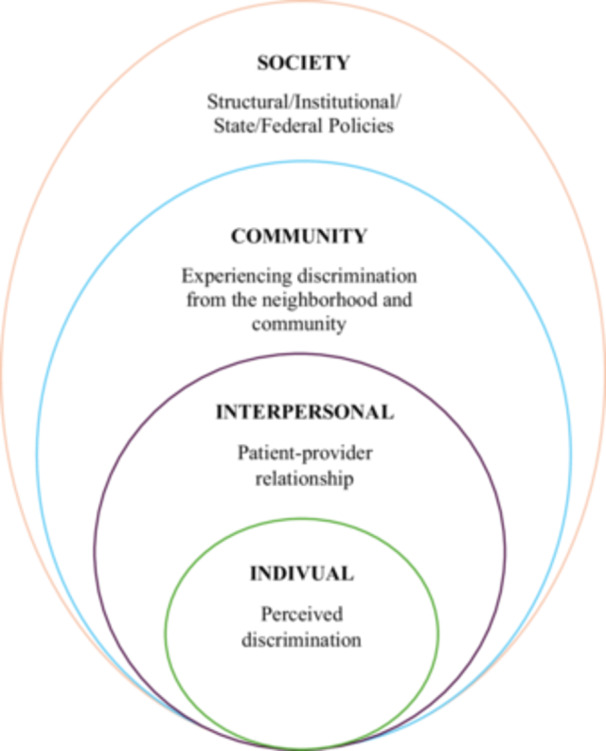
Diagram of the social ecological model applied to discrimination experience and prenatal care utilization.

We synthesized the literature in line with the key components of the SEM (Bronfenbrenner 1979). In doing so, we grouped studies that examined the effect of discrimination experiences among ethnic and racial minority pregnant women on PNC utilization into the various levels highlighted above (see Figure 1). This also meant that some studies could be grouped under more than one level, depending on the findings highlighted in the particular study considered. The application of the SEM provides a strategic foundation for identifying critical intervention points to effectively address and mitigate disparities in PNC utilization.

### 2.2 Study Design and Framework

We adopted the Population, Exposure, Comparator, and Outcome (PECO) framework, an adaptation of the Population, Intervention, and Outcome (PICO) model suitable for observational studies, to guide the current study (Morgan et al. 2018). PRISMA guidelines were used to guide the structure and reporting of the review (Page et al. 2021; Stroup 2000). The population included ethnic and racial minorities in the U.S., specifically Hispanic or Latino, Black or African American, American Indian and Alaska Native, Asian, Native Hawaiian, and Other Pacific Islander groups. The exposure of interest was discrimination, conceptualized through the lens of the SEM. The outcome was PNC utilization, which we operationalized to include frequency of visits or attendance, experiences with care, satisfaction, and the quality of PNC received. Although PNC utilization is commonly defined in terms of the number of PNC visits (Kotelchuck 1994), our broader operationalization was intended to capture the varied ways this concept is referenced across different studies. It also allowed us to go beyond quantitative evaluation of clinic attendance to include qualitative aspects of care. Where applicable, studies comparing different levels or types of discrimination exposure (e.g., high vs. low perceived discrimination) served as the basis for comparators. The study protocol was developed following the Methodological Expectations of Cochrane Intervention Reviews (MECIR) (Higgins et al. 2016) and was registered with PROSPERO (CRD420251012205).

### 2.3 Search Methods

In consultation with an institutional librarian and guided by the Peer Review of Electronic Search Strategies (PRESS) (McGowan et al. 2016), sample of detailed search strategy is provided in File S1. Although the PECO framework guided the development of our review question and conceptual approach, the population component was not explicitly represented in the search terms. This decision was purposeful to ensure a broad search strategy. We aimed to capture all potentially relevant studies, including those where the target population was not clearly identified in the title, abstract, or keywords but could be determined through full‐text screening. This approach prioritized sensitivity over specificity in the initial search phase, allowing for a more comprehensive identification of applicable literature.

### 2.4 Inclusion/Exclusion Criteria

Studies conducted between 2010 and 2024 and published in English (for ease of comprehension) were included. The year limit was set to ensure that findings reflect the contemporary relevance of the research question. Additionally, studies were selected based on their alignment with our defined PECO framework. However, studies whose findings were not directly related to the objective of this review were excluded. A sample of excluded studies and their rationale is attached as File S2.

Further, only studies conducted within the U.S. were considered to ensure the results were most relevant to the research topic and question. While discrimination in PNC is a global issue, this review focuses exclusively on studies conducted in the U.S. to ensure contextual consistency in how structural inequities, healthcare systems, and racial/ethnic classifications operate. The U.S. healthcare system is unique in its lack of universal PNC access and long‐standing racialized disparities in maternal health outcomes. Limiting the review to this context allows for clearer comparisons across studies and enables findings to be directly applicable to U.S.‐based policy and practice interventions. While important insights exist in other Western health systems, synthesizing those alongside U.S.‐based studies may have introduced heterogeneity that could obscure specific structural patterns relevant to the U.S. setting.

### 2.5 Data Selection

A total of 342 articles were initially identified for this review. The database search yielded 336 articles across the three selected databases. Additionally, six more studies were identified through Google Scholar and by reviewing article citations through a backward mapping process. All articles were managed using *Covidence software* (Veritas Health Innovation n.d), which facilitated the initial deduplication of 72 articles. The corresponding author (AM) manually verified these removals to ensure accuracy. The remaining 264 articles were title and abstract screened (by AM and AP), resulting in the exclusion (by consensual agreement) of 238 studies that were either irrelevant to the research question or conducted outside the United States. We (AM and AP) performed full‐text screening on 26 articles to assess alignment with the study's methodology, outcomes, and context. Of these, 21 were excluded by consensus: two were relevant but conducted outside the U.S., and 19 did not align with the research objective. This process resulted in a total of 11 studies included in this review.

### 2.6 Data Extraction

We created an Excel sheet that was used as an extraction template to record the details of studies that were considered. Key details of extraction included: authors/year of publication, aim of study, study design/methods, sample size, factors affecting PNC utilization, and SEM classification. Factors identified potentially overlaped in more than one of the SEM classifications. The final data set was reviewed jointly (by AM and AP) to ensure completeness and accuracy. Discrepancies during screening, appraisal, or data extraction were resolved through discussion and consensus. If consensus was not reached, a third author adjudicated the decision.

### 2.7 Data Synthesis

We conducted a quality appraisal of each of the included studies. Due to substantial heterogeneity across study designs (including qualitative, quantitative, and mixed methods), populations, and outcome measures, a meta‐analysis was not feasible. Variation in methodological frameworks, measurement tools, and definitions of both discrimination and PNC utilization limited the comparability of data across studies. These inconsistencies, combined with culturally embedded factors and context‐dependent barriers, necessitated an interpretive synthesis that could accommodate both narrative and statistical insights. Therefore, we synthesized, integrated, and narratively presented findings.

### 2.8 Quality Appraisal

The quality appraisal of included studies was assessed using the Joanna Briggs Institute (JBI) critical appraisal tools for analytical (quantitative) cross‐sectional studies (2017a) and for qualitative studies (2017b), respectively. Each study was independently reviewed by two authors (AM and AP) of this study. Discrepancies were resolved through discussion and consensus. The JBI checklist for both analytical cross‐sectional and qualitative studies evaluates several domains (10 for qualitative and 8 for quantitative), presented as footnotes under Table 2. Studies with mixed methods were assessed with both quantitative and qualitative checklists to help ensure each of the methods applied was sound. Each item was scored as “*Yes* = 1,” “*No* = 0,” “*Unclear* = 0,” or “*Not applicable* = 0.” For analytical cross‐sectional studies, overall scores were categorized as 0–2 (low quality: major limitations in design or reporting), 3–5 (moderate quality: some methodological issues), and 6–8 (high quality: reliable and well‐conducted). The qualitative studies’ scores ranged from 0 to 4 (low quality: significant methodological concerns), 5–7 (moderate quality: some limitations, but acceptable), and 8–10 (high quality: strong methodological rigor). This dual‐tool approach ensured that each study was evaluated using criteria appropriate to its design and informed the overall interpretation of evidence quality across the review.

## 3 Results

**Figure 2:**
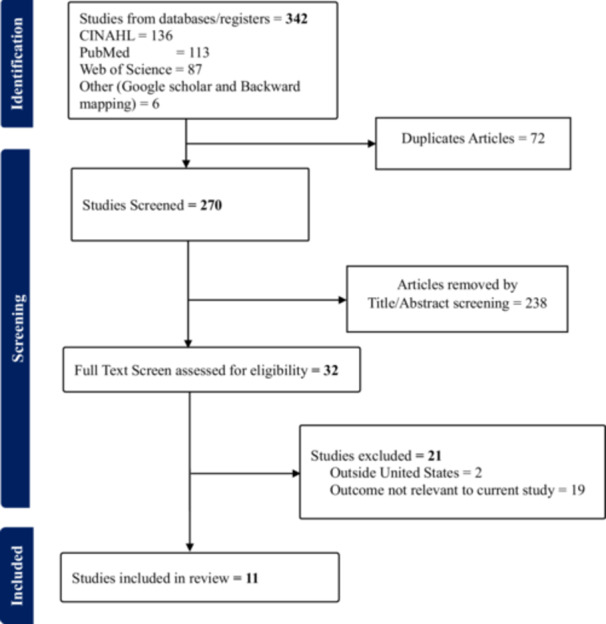
PRISMA flowchart of the literature search and study selection process.

Figure 2 below is a PRISMA flow diagram of the literature search and study selection process, showing the schematic presentation of the number of hits, exclusions, and final articles considered for this review.

### 3.1 Study and Participants’ Characteristics

This review synthesized findings from 11 peer‐reviewed studies published between 2010 and 2024, including Hispanic women, non‐Hispanic Black (African American) women, and Pacific Islanders in the U.S. (Table 1). The sample sizes range from small, in‐depth qualitative studies (e.g., Fryer et al. (2021), *n* = 11) to large‐scale quantitative analytical studies (e.g., Gillespie and Weeks (2021), *n* = 2571; Slaughter‐Acey et al. (2019), *n* = 1410). Dahlem et al. (2015) uniquely included both PNC recipients (*n* = 204) and providers (*n* = 21), reflecting a broader perspective on care experiences.

**Table 1:** Summary of included studies.

Author, year	Aim of study	Method	Sample	Key findings	Framework category
Teal et al. (2024)	Examine how racism, discrimination, and contextualized stress influence Black birthing people's preference for racially concordant prenatal care providers.	Quantitative	200 Black birthing women	Higher discrimination experience is linked to valuing racial concordance with prenatal providers. A positive correlation (r = 0.325, *p* = 0.007) exists between discrimination scores and the importance of racial concordance.	Interpersonal
Fryer et al. (2021)	To explore the barriers Spanish‐speaking women encounter in accessing quality prenatal care and identify factors that facilitate timely care.	Qualitative	11 Spanish‐speaking women	Latinx women face barriers like language challenges, lack of cultural competency, and ethnic discrimination. Racism and perceived discrimination, including undocumented status, hindered prenatal care utilization.	Structural (societal)
Reid et al. (2021)	Identified barriers and facilitators to early prenatal care reported by women in Florida.	Mixed method	55 Hispanic, non‐Hispanic white, and non‐Hispanic Black	Barriers faced by Hispanic, non‐Hispanic White, and non‐Hispanic Black birthing individuals in Florida, regarding PNC utilization include personal factors (e.g., mental health, pregnancy awareness, abortion), community challenges (e.g., transportation, stigma, fear, limited social support), and healthcare system issues (e.g., language barriers, clinic delays, cost of care).	Intrapersonal level Structural (Societal)
Gillespie and Weeks (2021)	Examined nonwhite women's experience of racism in pre and postanal care utilization.	Quantitative	2571 nonwhite Mothers	Less‐than‐adequate prenatal care is significantly associated with racial discrimination (OR: 1.4; 95% CI: 1.02–1.8). After adjusting for sociodemographic factors, the relationship becomes marginally significant (OR: 1.3; 95% CI: 0.9–1.7).	Interpersonal
Dove‐Medows et al. (2021)	To provide a qualitative analysis of Black women's experiences with racial discrimination during pregnancy to highlight its complexities.	Qualitative	18 Black pregnant women	Women reported diverse experiences of racial discrimination in various contexts. Shielding was a common coping strategy for dealing with racial discrimination during pregnancy.	Intrapersonal
Dillon et al. (2020)	To examine discriminatory factors contributing to prenatal care dissatisfaction and disengagement among young expecting couples, with a focus on Black and Latinx experiences.	Quantitative	296 Black and Latinx Adolescent and Young Couples	Frequent healthcare discrimination increases (3.1 times) unpleasant prenatal care experiences (95% CI: 1.60–5.08) compared to those who experienced discrimination at most once a year or never. Language‐related discrimination leads to fewer prenatal care visits (*p* = 0.04).	Interpersonal Structural (Societal)
Slaughter‐Acey et al. (2019)	Investigate how perceived discrimination, specifically racial microaggressions, contributes to delayed prenatal care among African American women.	Quantitative	1410 Black, African American women	Higher Daily Life Experiences of Racism and Bother scores are linked to delayed prenatal care (AOR = 1.31, 95% CI = 1.00, 1.71). Maternal skin tone moderates this relationship. Delayed prenatal care is more common for African American women with light or dark brown skin tones, but not for medium brown women.	Community level
Ayers et al. (2018)	Examines Marshallese mothers’ beliefs, perceptions, and experiences of prenatal care while identifying potential barriers to access and utilization.	Qualitative	43 Pacific Islanders	Major structural barriers included health insurance, transportation, and language issues. Participants reported perceived discrimination from prenatal care providers. Fear was also a significant barrier to seeking prenatal care.	Intrapersonal level Structural (Societal)
D'Angelo et al. (2016)	Explores prenatal care improvements in Connecticut to address racial and ethnic disparities, as Black and Hispanic infant mortality rates exceed the state average.	Qualitative	47 Hispanic and Black/African American women	Women appreciated providers’ advice but felt unheard and faced structural challenges, indicating a need for policy changes. While racial discrimination was not reported, some women experienced other forms of discrimination in prenatal care.	Structural (Societal)
Dahlem et al. (2015)	To determine African American women's experiences with patient‐provider interactions, focusing on communication, perceived discrimination, and their impact on prenatal care perceptions and health behaviors.	Quantitative	204 African American women and 21 providers	Women experienced good communication with their healthcare providers and encountered minimal discrimination. Effective patient‐provider communication improved trust in providers (*β* = 0.75, *p* < 0.001) and prenatal care satisfaction (*β* = 0.81, *p* < 0.001), but not adherence to prenatal health behaviors.	Interpersonal
Slaughter‐Acey et al. (2013)	Examines how personal and group racism influences prenatal care entry among low‐income African American women and whether denying racism delays care access.	Quantitative	872 African American Women	Denial of racism by others hindered early prenatal care access for low‐income African American women (AOR = 1.19; 95%CI: 1.00–1.41; *p* = 0.05). Delayed prenatal care may stem from women avoiding racialized experiences and discrimination due to a lack of empowerment.	Community level

**Figure 3:**
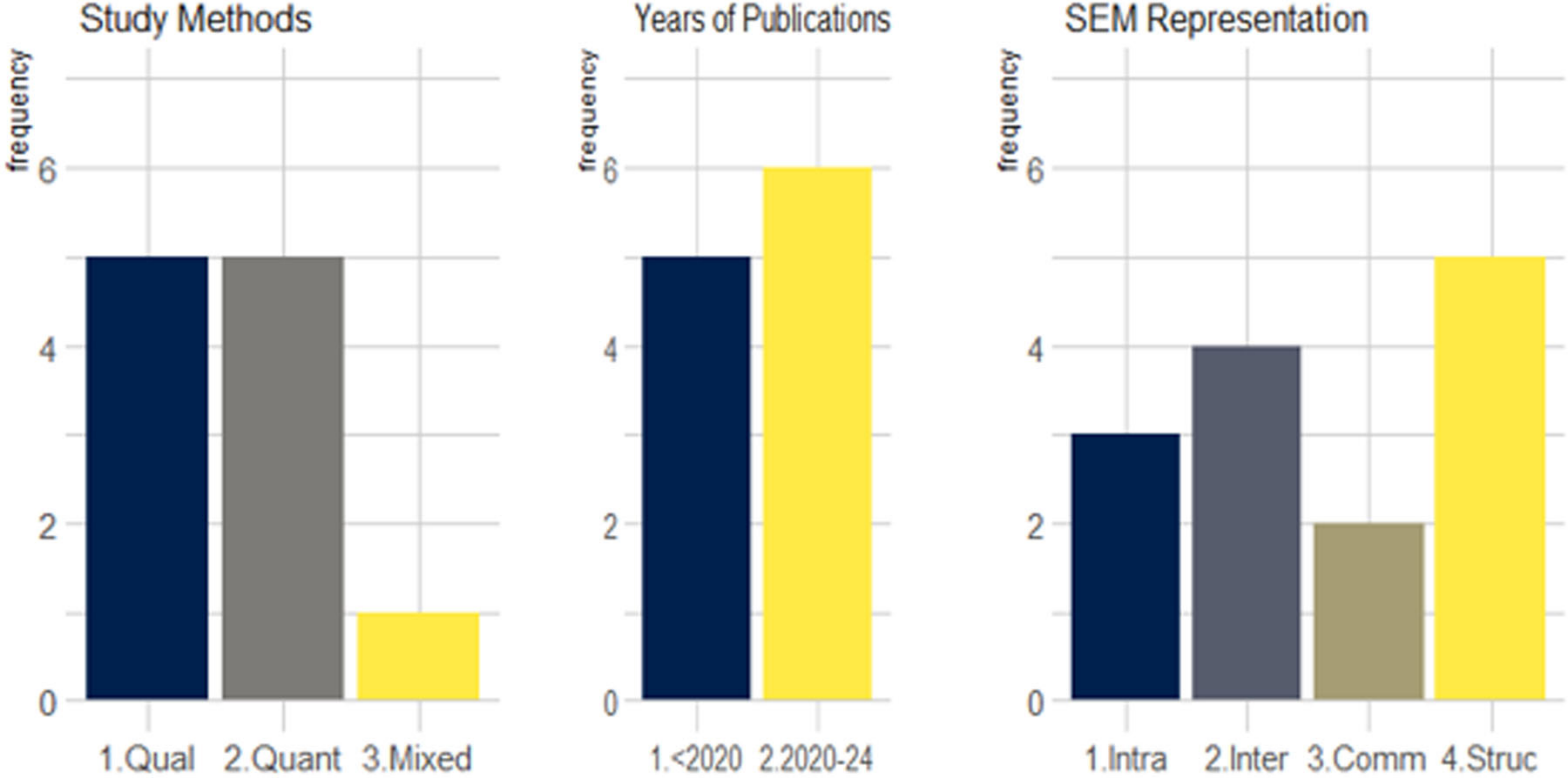
Summary of study methods, years of publication, and socio‐ecological model representation.

Across the 11 included studies, five used quantitative methods (Dillon et al. 2020; Gillespie and Weeks 2021; Slaughter‐Acey et al. 2013, 2019; Teal et al. 2024), another five used qualitative methods (Ayers et al. 2018; Dahlem et al. 2015; Dove‐Medows et al. 2021; Fryer et al. 2021; Reid et al. 2021), while one used mixed methods (D'Angelo et al. 2016). The majority (6/11) of studies were conducted between 2020 and 2024, with most of them (5/11) highlighting structural barriers (see Figure 3).

### 3.2 Quality Appraisal Summary

From Table 2, all qualitative studies scored 8/10, indicating consistent methodological rigor. The domains most frequently unmet were D6 (researcher's cultural/theoretical position) and D7 (researcher's influence on the research), which were rated as “No” across all studies. Despite these omissions, all qualitative studies were rated as “High Quality,” reflecting strong congruity in philosophical alignment, data collection, and interpretation. For the quantitative studies, four achieved a perfect score of 8/8, while two scored 6/8, primarily due to unmet criteria in D5 (strategies to deal with confounding) and D6 (valid and reliable outcome measurement). All studies were rated as “High Quality,” indicating robust methodological design and appropriate statistical analysis. D'Angelo et al. (2016) was appraised both quantitatively and qualitatively based on its mixed‐methods approach.

**Table 2:** Quantitative and Qualitative Studies Assessed using the JBI checklist.

Qualitative studies	D1	D2	D3	D4	D5	D6	D7	D8	D9	D10	Score	Overall quality
Fryer et al. (2021)	Yes	Yes	Yes	Yes	Yes	No	No	Yes	Yes	Yes	8/10	High Quality
Reid et al. (2021)	Yes	Yes	Yes	Yes	Yes	No	No	Yes	Yes	Yes	8/10	High Quality
Dove‐Medows et al. (2021)	Yes	Yes	Yes	Yes	Yes	No	No	Yes	Yes	Yes	8/10	High Quality
Ayers et al. (2018)	Yes	Yes	Yes	Yes	Yes	No	No	Yes	Yes	Yes	8/10	High Quality
D'Angelo et al. (2016)	Yes	Yes	Yes	Yes	Yes	No	No	Yes	Yes	Yes	8/10	High Quality
Dahlem et al. (2015)	Yes	Yes	Yes	Yes	Yes	No	No	Yes	Yes	Yes	8/10	High Quality
**Quantitative (analytical) Studies**	**D1**	**D2**	**D3**	**D4**	**D5**	**D6**	**D7**	**D8**				
Teal et al. (2024)	Yes	Yes	Yes	Yes	No	No	Yes	Yes			6/8	High Quality
Dillon et al. (2020)	Yes	Yes	Yes	Yes	Yes	Yes	Yes	Yes			8/8	High Quality
D'Angelo et al. (2016)	Yes	Yes	Yes	Yes	No	No	Yes	Yes			6/8	High Quality
Gillespie and Weeks (2021)	Yes	Yes	Yes	Yes	Yes	Yes	Yes	Yes			8/8	High Quality
Slaughter‐Acey et al. (2019)	Yes	Yes	Yes	Yes	Yes	Yes	Yes	Yes			8/8	High Quality
Slaughter‐Acey et al. (2013)	Yes	Yes	Yes	Yes	Yes	Yes	Yes	Yes			8/8	High Quality

*Note:* Quality Appraisal Using Joanna Briggs Institute (JBI) Checklist for Cross‐Sectional Analytical and Qualitative Methods. D: Domain.

Qualitative Checklist.

D1: Is there congruity between the stated philosophical perspective and the research methodology?

D2: Is there congruity between the research methodology and the research question or objectives?

D3: Is there congruity between the research methodology and the methods used to collect data?

D4: Is there congruity between the research methodology and the methods used to collect data?

D5: Is there congruity between the research methodology and the interpretation of results?

D6: Is the researcher's cultural or theoretical position stated?

D7: Is the influence of the researcher on the research, and vice versa, addressed?

D8: Are participants, and their voices, adequately represented?

D9: Is the research ethical according to current criteria, and is there evidence of ethical approval by an appropriate body?

D10: Do the conclusions drawn in the research report flow from the analysis or interpretation of the data?

Quantitative Checklist.

D1: Clear inclusion criteria.

D2: Valid methods for condition identification.

D3: Exposure measured reliably.

D4: Confounding factors identified.

D5: Strategies to deal with confounding.

D6: Outcome measured validly and reliably.

D7: Appropriate statistical analysis.

### 3.3 Discrimination (Intrapersonal Level) and PNC Utilization

Most studies reported that individuals who felt discriminated against were less likely to utilize PNC. This finding highlights how individual factors hinder PNC utilization. For example, Reid et al. (2021) found that the fear of internalized discrimination was a key factor affecting the early initiation of PNC among Hispanic, non‐Hispanic White, and non‐Hispanic Black women in Florida. While the sample included non‐Hispanic White participants as well, these findings are relevant to racial and ethnic minorities within the study population, though not reported separately. Similarly, a community‐based study by Ayers et al. (2018) noted that participants’ previous experiences of discrimination from healthcare providers influenced their perceptions and ultimately hindered their PNC visits.

A qualitative study by Dove‐Medows et al. (2021) revealed that some pregnant women of Black race often chose to avoid PNC to escape racial discrimination. These findings illustrate how the intrapersonal factor, perceived discrimination, can influence PNC utilization.

### 3.4 Discrimination (Interpersonal Level) and PNC Utilization

Statistical analysis demonstrated that negative patient‐provider relationships, interpreted as racial discrimination, from healthcare providers negatively affected PNC utilization (Gillespie and Weeks 2021). This was illustrated by a marginally significant association between racial discrimination and fewer PNC visits compared to the recommended guidelines (American Academy of Pediatrics & American College of Obstetricians and Gynecologists 2017).

Also, Dillon et al. (2020) noted that experiencing discrimination from healthcare providers affected PNC's satisfaction. Teal et al. (2024) found that experiences of racism and discrimination significantly compromised Black birthing people's engagement with PNC. They reported how discriminatory interactions with healthcare providers led to diminished trust, reduced satisfaction, and hesitancy in attending prenatal appointments. These experiences often resulted in delayed or avoided care, with some individuals expressing a preference for racially concordant providers to mitigate the emotional toll and improve communication. The study underscores that perceived discrimination may indirectly affect the quality and continuity of PNC utilization.

### 3.5 Discrimination (Community Level) and PNC Utilization

Some studies reported how discrimination at this level affects PNC utilization, particularly visits (Slaughter‐Acey et al. 2019). For instance, racial microaggressions (subtle, often unintentional behaviors or comments that convey bias or stereotypes toward people based on their race or ethnicity) were observed among Black/African American women, which statistically influenced adequate PNC utilization (Slaughter‐Acey et al. 2019). In their study, Slaughter‐Acey et al. (2019) demonstrated that *Daily Life Experiences of Racism and Bother Scale (DLERBS)* scores above the median were associated with delayed PNC visits. Thus, a higher *DLERBS* score was linked to delayed PNC visits among African American women.

Additionally, using the *Racism and Life Experience Index*, Slaughter‐Acey et al. (2013) reported a relationship between group racism (i.e., the experience of racism by close relations, family, friends, neighbors, etc.) and late or no PNC visits among African American women. The authors further contend that delayed utilization of PNC may stem from the avoidance of racialized experiences among less‐empowered women, suggesting that feeling less empowered makes navigating PNC difficult.

### 3.6 Discrimination (Structural/Societal Level) and PNC Utilization

Some studies reported that structural issues presenting as discriminatory vary and influence PNC utilization among minority pregnant women. For instance, Spanish‐speaking women in a qualitative study identified language barriers as part of the structural discrimination challenges they face when navigating PNC (Fryer et al. 2021). Specifically, they encountered longer waiting hours due to a lack of timely interpreter services.

Similarly, Dillon et al. (2020) noted that Black and Latinx young adolescents considered language barriers a form of discrimination, and those who experienced such barriers statistically attended fewer PNC visits compared to those who did not. Additionally, Reid et al. (2021) and Ayers et al. (2018) identified language as a structural/healthcare system barrier to PNC utilization in Florida and among Marshallese mothers in Arkansas, respectively.

A study by D'Angelo et al. (2016) aimed to highlight the influence of social determinants of health on PNC utilization. It found that some pregnant women reported experiencing discrimination plausibly linked to institutional policies—particularly feelings of being unheard during their prenatal care experiences, such as a lack of consideration for personal preferences and language barriers.

## 4 Discussion

This review highlights the multifaceted aspects of discrimination and its effect on PNC utilization, aligning with the theoretical framework of the Social‐Ecological Model (Bronfenbrenner 1979). Despite the critical importance of this topic, the body of research includes only 11 studies identified over the past 14 years, predominantly focusing on non‐Hispanic Black (African American) and Hispanic women in the U.S. context.

The relationship between discrimination (at various levels of the SEM) and PNC utilization among ethnic/racial minority populations is evident, often resulting in delays or complete avoidance of care (Dove‐Medows et al. 2021; Slaughter‐Acey et al. 2013). These findings align with established literature and theoretical perspectives on structural racism, highlighting systemic barriers that exacerbate inequities in PNC utilization (Alhusen et al. 2016; Carter et al. 2016; Hadley et al. 2023; Viruell‐Fuentes et al. 2012). This alignment is particularly concerning, given the established relationship between inadequate or delayed PNC and heightened maternal mortality risks (Gadson et al. 2017).

Notably, most studies have focused on internalized factors, including fear of and perceived discrimination. This underscores the need to understand how positive individual‐level factors (e.g., resilience) and interpersonal factors (e.g., social support) may buffer the adverse effects of discrimination on PNC utilization. While the SEM provides a valuable framework for identifying the multiple levels of influence on health behaviors, it is primarily structural and descriptive. It outlines where influences occur but does not fully explain how or why these influences affect health outcomes, particularly among marginalized populations.

To address this gap, future studies can also consider the Minority Stress Theory (MST) (Meyer 2003) to offer a complementary and more explanatory framework. Although MST was not used to guide the present review, its application in future studies could enrich the understanding of the psychological and social pathways linking discrimination to PNC use. This complementary perspective may support the development of more targeted and equity‐informed interventions. MST, originally designed for sexual and gender minorities, posits that people from stigmatized social groups experience chronic stress due to discrimination, prejudice, and social disadvantage, which can negatively affect health behaviors and outcomes. Importantly, MST also emphasizes the role of protective factors, including resilience and social support, that can mitigate the harmful effects of minority stress. Incorporating MST into this line of inquiry allows for a more nuanced understanding of the psychological and social mechanisms through which discrimination affects PNC utilization. It also supports the development of targeted interventions that not only address risk but also strengthen resilience and support systems within marginalized communities. Together, SEM and MST provide a multidimensional foundation for future research and program development aimed at improving maternal health equity.

Additionally, this review noted the presence of discrimination in healthcare settings, particularly from healthcare providers, which significantly impedes PNC utilization among ethnic/racial minority pregnant women. For instance, African American and Hispanic young adults experienced high levels of racial discrimination and unpleasant PNC experiences from healthcare providers (Dillon et al. 2020). Similarly, provider discrimination led Black women to avoid PNC (Dove‐Medows et al. 2021). These findings imply that experiences of discrimination from healthcare providers tend to hinder compliance, thereby exacerbating the vulnerability of ethnic/racial minority pregnant women during pregnancy.

Building on evidence that discrimination fosters mistrust in healthcare systems and limits access to essential services (Bazargan et al. 2021; Carter et al. 2017), it is critical to explore actionable solutions proposed by recent studies. For instance, incorporating real‐time feedback mechanisms during and after prenatal visits can help address perceived discrimination and improve patient‐provider interactions, particularly for minority groups like African American and Hispanic mothers (Bruxvoort 2021; Dillon et al. 2020; Teal et al. 2024). Such feedback enables healthcare teams to identify and rectify issues early, reducing stress on expecting mothers and preventing disengagement from care.

Additionally, trauma‐informed, person‐centered approaches that aim to reduce the psychological and structural harm associated with discrimination can foster more supportive care environments (Dove‐Medows et al. 2021). These approaches center mothers as experts in their experiences, rather than placing the burden of risk mitigation solely on them. Maternal‐child nurses, in particular, should be trained to recognize and validate the lived experiences of ethnic/racial minority pregnant women, using tools such as the *Experiences of Discrimination Scale* (Krieger et al. 2005) to assess and address the impact of racism. These strategies, combined with ongoing cultural competence training for providers, can help mitigate biases and improve maternal and infant health outcomes.

Although only two studies have examined community‐level factors within the SEM, the findings, particularly that of Slaughter‐Acey et al. (2019), suggest that perceived racial microaggressions may contribute to delayed engagement with PNC. However, the current evidence in this area remains limited, making it difficult to fully understand the impact of community‐level discrimination on PNC utilization. Further research is needed to explore the specific dimensions of community‐level discrimination and their influence on PNC utilization among marginalized populations. Longitudinal studies could offer deeper insights into how experiences of discrimination shape PNC engagement over time. Additionally, examining the intersectionality of race, ethnicity, socioeconomic status, and other social determinants of health may provide a more nuanced understanding of the barriers faced by minority communities.

Language differences also present a particularly significant challenge for non‐English‐speaking populations among the discriminatory barriers, further exacerbating disparities in PNC utilization. Spanish‐speaking women and other minority groups often encounter limited translator availability and prolonged wait times, which hinder their ability to access timely and effective care (Ayers et al. 2018; Fryer et al. 2021; Reid et al. 2021). Addressing this structural challenge requires expanding language access solutions, such as on‐demand interpretation services and telemedicine‐based translation tools, to improve communication and reduce delays. Real‐time translation during appointments and increased interpreter availability can enhance patient‐provider interactions. Hiring bilingual staff and implementing cultural competency training for healthcare providers can create more inclusive care environments. Patient‐centered approaches, including multilingual educational materials and language‐sensitive appointment scheduling systems, can further facilitate access to PNC. Community‐based interventions, such as mobile clinics and community health workers, may also help bridge gaps in care for non‐English‐speaking pregnant individuals. These measures address logistical barriers and mitigate the psychological burden of navigating biased systems, as evidenced by the “shielding” phenomenon—staying away from care to avoid the experience of discrimination (Dove‐Medows et al. 2021). By proactively addressing language barriers, healthcare systems can reduce disparities and improve PNC access for marginalized populations.

Beyond Black and Hispanic populations, Southeast Asian and Indigenous women are less likely to attend prenatal check‐ups and postnatal follow‐ups, with their disengagement often stemming from alienation and mistrust of the healthcare system (Han 2024). This increases the risk of adverse health outcomes for both mothers and infants. Southeast Asian immigrant women, particularly those with limited English proficiency, often encounter provider bias, cultural insensitivity, and systemic barriers that deter them from seeking timely care (Vo 2023). Indigenous women, meanwhile, report mistrust in healthcare systems due to historical trauma and ongoing disparities in provider communication and quality of care (Holder 2024). These intersecting factors highlight the necessity of culturally and linguistically responsive institutional structures. Addressing systemic challenges including language barriers, prolonged waiting times, and culturally discordant care is essential for improving PNC utilization across diverse minority populations. A more intersectional approach that considers race, ethnicity, language, immigration status, and socioeconomic factors is critical for dismantling barriers and promoting equitable access to care.

Although this review focused on studies conducted in the U.S., its insights have broader global relevance. In many parts of the world, disadvantaged populations, including racial, ethnic, linguistic, and socioeconomic minorities, experience comparable forms of discrimination that impede access to PNC. Therefore, understanding how multilevel inequities shape prenatal care utilization in the U.S. context can inform cross‐national efforts to promote maternal health equity and culturally responsive care models worldwide.

### 4.1 Implications from the Review

This review underscores the significant impact of discrimination on PNC utilization among ethnic/racial minority populations, highlighting the need for healthcare providers, particularly those in maternal‐child healthcare, to be culturally sensitive and proactive in promoting equitable care. Implementing cultural competence training that includes trauma‐informed principles, antiracism education, and simulations addressing implicit bias is essential for improving provider‐patient interactions. To assess and respond to patients’ experiences, nurses can use validated tools such as the *Experiences of Discrimination Scale* (Krieger et al. 2005), which helps capture perceptions of bias in clinical settings. This also underscores the need for conceptual clarity (a concept analysis) regarding the concept of healthcare utilization, particularly in maternal and child health research. A comprehensive definition that will align with evolving global health priorities and quality‐of‐care frameworks, extending beyond the number of prenatal or postnatal visits to include qualitative dimensions of care, such as respectful care, autonomy, continuity, and cultural adaptability.

Additionally, fostering positive patient‐provider relationships requires consistent self‐reflection and responsiveness to feedback, which may include multilingual patient surveys and real‐time communication assessments. Maternal‐child nurses should also connect patients with culturally aligned support systems and advocate for institutional reforms, such as on‐demand interpretation services, to improve access for linguistically diverse populations. In addition, nurse‐led research and policy engagement are vital for addressing structural challenges and informing sustainable changes in care delivery. By integrating these evidence‐informed practices, healthcare providers can help reduce disparities and enhance PNC utilization across marginalized communities.

Beyond nursing, the findings have broader policy implications, especially in shaping guidelines for PNC delivery. Policymakers should consider investing in community health worker programs, expanding Medicaid coverage for prenatal services, and standardizing cultural competence protocols in federally funded clinics. Additionally, maternity care incentives could be tied to equity benchmarks and language access compliance. By adopting these integrated strategies, healthcare professionals, researchers, and policymakers can work together to create inclusive systems of care that promote equitable outcomes for all pregnant individuals.

### 4.2 Limitations of the Study

Several limitations of this study need to be acknowledged. For instance, the review did not include an analysis of policy documents related to structural factors, which may have provided important contextual information. Although a thorough search strategy and varied databases were used, the findings may not be exhaustive. Study categorization by SEM levels relied on the authors’ framing, which may differ if appraised by others, acknowledging potential overlap between levels. Also, due to heterogeneity in study designs and measures, and the broader scope and meaning of utilization, a meta‐analysis was not feasible.

## 5 Conclusion

Overall, there is available evidence suggesting that discrimination negatively affects the utilization of prenatal care among ethnic/racial minority populations in the United States. Further evidence from varied settings and larger samples is recommended to strengthen the evidence base and clarify this relationship. Longitudinal studies could provide valuable insights into the long‐term effects of discrimination on maternal and infant health outcomes. Additionally, future research should explore the intersectionality of race, ethnicity, socioeconomic status, and other social determinants of health to capture how these factors influence PNC utilization.

This systematic review also underscores the need to examine the buffering effects of other intrapersonal and interpersonal factors (as outlined in the SEM), such as resilience and social support, in mitigating the impact of discrimination on PNC utilization. Such research is particularly important for establishing a baseline for future intervention studies aimed at increasing PNC utilization among ethnic and racial minorities. Notwithstanding the gaps, addressing negative patient‐provider relationships and systemic or structural challenges, including language barriers is essential to promoting equitable PNC utilization. As the healthcare system evolves, prioritizing the needs of minority populations will be key to fostering a more inclusive and supportive environment for minority pregnant women, ultimately enhancing maternal and infant health outcomes and reducing disparities.

## Author Contributions

Abdul‐Manaf Mutaru, Alexa Parra, Cynthia Nicole Lebron, Wonsuk Yoo, and Huson P. Santos Jr made substantial contributions to conception and design, or acquisition of data, or analysis and interpretation of data. Abdul‐Manaf Mutaru, Alexa Parra, Cynthia Nicole Lebron, Wonsuk Yoo, and Huson P. Santos Jr involved in drafting the manuscript or revising it critically for important intellectual content. Abdul‐Manaf Mutaru, Alexa Parra, Cynthia Nicole Lebron, Wonsuk Yoo, and Huson P. Santos Jr given final approval of the version to be published. Each author should have participated sufficiently in the work to take public responsibility for appropriate portions of the content. Abdul‐Manaf Mutaru, Alexa Parra, Cynthia Nicole Lebron, Wonsuk Yoo, and Huson P. Santos Jr agreed to be accountable for all aspects of the work in ensuring that questions related to the accuracy or integrity of any part of the work are appropriately investigated and resolved.

## Consent

This study did not involve interaction with patients, service users, caregivers, or members of the public.

## Supporting information


**Supplentary File 1:** Search Strategy.

**Supplementary File 2:** Excluded Studies.

PRISMA Checklist.

## Data Availability

The data that support the findings of this study are available from the corresponding author upon reasonable request.
