# Supplementary material for: Examining the Role of Discrimination in Prenatal Care Utilization: A Systematic Review Using the Social‐Ecological Model
Source: Res Nurs Health. 2025 Nov 16;49(1):60–73. doi: 10.1002/nur.70033 (PMC12779230; doi:10.1002/nur.70033)
Supplement: Supplementary file 1 — Supplentary File 1: Search Strategy. [file NUR-49-60-s003.pdf]

Databases Searched

- Cumulative Index to Nursing and Allied Health Literature (CINAHL)
- PubMed
- Web of Science

Search Dates: November 1, 2024.

Search Strings with Boolean Logic (PubMed):

("social determinants of health"[All Fields] OR "Perceived discrimination"[All Fields] OR "racial bias"[All Fields] OR "race/ethnicity"[All Fields] OR "social inequality"[All Fields] OR "ethnic discrimination"[All Fields] OR "prejudice"[All Fields] OR "Implicit bias"[All Fields] OR "Explicit bias"[All Fields] OR "racism"[All Fields]) AND ("prenatal care"[All Fields] OR "first trimester"[All Fields] OR "Antenatal care"[All Fields] OR "pregnanc*"[All Fields]) AND ("Healthcare utilization"[All Fields] OR "barriers to care"[All Fields] OR "Health Care Acceptability"[All Fields] OR "Health Care Seeking Behavior"[All Fields] OR "Patient participation"[All Fields] OR "Health care system"[All Fields]).

Results Retrieved = 113 articles

Filters Applied

- Articles published in English language
- Peer-reviewed articles
- Publication date: 2010 – 2024
- Research conducted in the United States
