# Supplementary material for: Examining the Role of Discrimination in Prenatal Care Utilization: A Systematic Review Using the Social‐Ecological Model
Source: Res Nurs Health. 2025 Nov 16;49(1):60–73. doi: 10.1002/nur.70033 (PMC12779230; doi:10.1002/nur.70033)
Supplement: Supplementary file 2 — Excluded Studies. [file NUR-49-60-s001.docx]

**Supplementary File 2:**

*Sample of Excluded Studies*

| **Authors (Year)** | **Aim** | **Finding** | **Reason for Exclusion** |
| --- | --- | --- | --- |
| Attanasio et al. (2018)  [doi.org/10.1016/j.pec.2018.01.002](https://doi.org/10.1016/j.pec.2018.01.002) | To examine correlates of shared decision making during labor and delivery. | Black women in marginalized social groups are less likely to report shared decision-making during birth/delivery. | Exposure measures shared decision making, and the outcome also focuses on birth/delivery instead of prenatal care. |
| Curtis et al. (2024)  [doi.org/10.1016/j.healthplace.2024.103177](https://doi.org/10.1016/j.healthplace.2024.103177) | Developed county-level measures of care barriers and analyzed their links to birth outcomes among U.S.-born Black and White mothers (2014–2017). | Structural barriers were not linked to small-for-gestational-age births, and barriers specific to Black residents showed no association with White birth outcomes, except for the Black uninsurance rate. | Outcome is focused on birth outcomes instead of prenatal care. |
| Flanagan et al. (2018)  [doi.org/10.1089/jwh.2017.6649](https://doi.org/10.1089/jwh.2017.6649) | To evaluate the feasibility and acceptability of screening for ACEs in standard prenatal care. | Clinicians' willingness to screen for ACEs was contingent on adequate training, streamlined workflows, inclusion of resilience screening, and availability of mental health, parenting, and social work resources. | Exposure measures adverse childhood experiences.  The population is not ethnic/racial minority. |
| Grytten et al. (2011)  [doi.org/10.1016/j.jhealeco.2010.10.004](https://doi.org/10.1016/j.jhealeco.2010.10.004) | To address models that can explain why expert patients (obstetricians, midwives, and doctors) are treated better than non-experts (mainly non-medical training). | Models of statistical discrimination show that benevolent doctors treat expert patients better, since experts are better at communicating with the doctor. | Exposure, population, and outcome are inconsistent with the current review. |
| Hall et al. (2020)  [doi.org/10.1007/s10995-020-02941-3](https://doi.org/10.1007/s10995-020-02941-3) | Examined racial disparities in prenatal care use and infant SGA among active-duty U.S. military women, a population with equal healthcare access and known socioeconomic status. | Across multiple assessments, non-Hispanic Black military women had consistently worse prenatal care use and infant SGA outcomes than non-Hispanic White women. | Stratified analysis, rather than examining prenatal care as an outcome among the population and discrimination as exposure. |
| James et al. (2023)  [doi.org/10.1111/birt.12755](https://doi.org/10.1111/birt.12755) | Examined the link between discrimination during childbirth hospitalization and postpartum care use among Black birthing people in California. | Postpartum care use among Black birthing people in California is influenced by complex factors. | Outcome focused on delivery and postpartum care, instead of prenatal care. |
